# Paving Behavior in Ants and Its Potential Application in Monitoring Two Urban Pest Ants, *Solenopsis invicta* and *Tapinoma melanocephalum*

**DOI:** 10.3390/insects14030219

**Published:** 2023-02-23

**Authors:** Liming Shen, Chao Wen, Xuan Chen, Yan Hua, Chengju Du, Jiacheng Cai, Xiujun Wen, Lei Wang, Cai Wang

**Affiliations:** 1 College of Forestry and Landscape Architecture, South China Agricultural University, Guangzhou 510642, China; 2 School of Grassland Science, Beijing Forestry University, Beijing 100083, China; 3 Department of Biological Sciences, Salisbury University, Salisbury, MD 21801, USA; 4 Guangdong Provincial Key Laboratory of Silviculture, Protection and Utilization, Guangdong Academy of Forestry, Guangzhou 510520, China; 5 Department of Mathematical Sciences, Salisbury University, Salisbury, MD 21801, USA; 6 College of Plant Protection, South China Agricultural University, Guangzhou 510642, China

**Keywords:** monitoring, pest ant, fire ant, ghost ant, adhesive tape

## Abstract

**Simple Summary:**

Adhesive tapes are as effective as baits and pitfall traps in monitoring *Solenopsis invicta* and *Tapinoma melanocephalum* in urban areas in southern China. However, adhesive tapes detected less ants and fewer species of non-target ants than baits and pitfall traps. Our study demonstrated that tape-paving behavior can potentially be used in developing more specific monitoring methods for *S. invicta* and *T. melanocephalum* in urban areas in southern China.

**Abstract:**

Our previous study discovered that two urban pest ants, red imported fire ants, *Solenopsis invicta* Buren (Formicidae: Myrmicinae), and ghost ants, *Tapinoma melanocephalum* (Fabricius) (Formicidae: Dolichoderinae), can pave viscose surfaces with particles to facilitate food search and transport. We hypothesize that this paving behavior can be applied to monitor *S. invicta* and *T. melanocephalum*. In the present study, 3998 adhesive tapes, each with a food source (sausage), were placed in 20 locations around Guangzhou, China (181–224 tapes per location), and their efficiency to detect *S. invicta* and *T. melanocephalum* was compared with two traditional ant-monitoring methods, baiting and pitfall trapping. Overall, *S. invicta* was detected by 45.6% and 46.4% of baits and adhesive tapes, respectively. In each location, the percentage of adhesive tapes detecting *S. invicta* and *T. melanocephalum* was similar when compared to baits and pitfall traps. However, significantly more non-target ant species showed up on bait and pitfall traps. Seven non-target ant species—*Pheidole parva* Mayr (Formicidae: Myrmicinae), *Pheidole nodus* Smith (Formicidae: Myrmicinae), *Pheidole sinica* Wu & Wang (Formicidae: Myrmicinae), *Pheidole yeensis* Forel (Formicidae: Myrmicinae), *Carebara affinis* (Jerdon) (Formicidae: Myrmicinae), *Camponotus nicobarensis* Mayr (Formicidae: Formicinae), and *Odontoponera transversa* (Smith) (Formicidae: Ponerinae)—also showed tape paving behavior, but they can be easily distinguished morphologically from *S. invicta* and *T. melanocephalum*. Our study showed that the paving behavior occurs in different subfamilies of ants (i.e., myrmicinae, dolichoderinae, formicinae, and ponerinae). In addition, paving behavior can potentially be used to develop more specific monitoring methods for *S. invicta* and *T. melanocephalum* in urban areas in southern China.

## 1. Introduction

The red imported fire ant, *Solenopsis invicta* Buren (Formicidae: Myrmicinae), is a globally distributed pest ant that threatens human health due to its highly aggressive behaviors and painful stings that can cause severe allergic reactions [1,2]. In many regions, *S. invicta* invasion has become a serious medical problem, especially in urban areas where humans encounter fire ants at high frequencies. According to a survey conducted by Xu et al. [3], more than one-third of people suffered painful stings in *S. invicta*-infested areas in China. Although Wang et al. [4] reported that fire ant stings might not significantly affect the mental health of city residents, a small portion of people stung by fire ants did meet the criteria for post-traumatic stress disorder.

Our previous studies showed that *S. invicta* has successfully invaded many green spaces (e.g., street gardens and green belts) and city parks in southern China [5,6]. Monitoring *S. invicta* can be challenging, particularly in urban areas. Fire ants often do not build their typical mound structures due to frequent rains, irrigation, and cleaning activities; therefore, mound-based monitoring methods may not have high efficiency to detect *S. invicta* in cities. In addition, though baits and pitfall traps provide valuable methods to collect fire ants, they attract many non-target ant species, and sorting out the collected ants is not a trivial task. Although rapid *S. invicta* detection methods based on loop-mediated isothermal amplification and lateral flow immunoassay have been developed [7,8], these technologies are not yet commercially available, probably due to their high prices and requirements for specific skills and equipment.

In a recent study, we found that *S. invicta* actively relocated particles to cover adhesive tapes [9]. We believe this behavior is closely associated with the foraging activities of *S. invicta* because: (1) the presence of food significantly increased the paved areas of the tape under both laboratory and field conditions; and (2) *S. invicta* could not successfully search for and transport food items on adhesive surfaces unless they paved the tapes with enough particles. We also recently observed similar tape-paving behaviors in *Tapinoma melanocephalum *(Fabricius) (Formicidae: Dolichoderinae), a serious urban ant pest that has invaded many tropical and subtropical regions worldwide [10,11,12,13]. Like *S. invicta*, *T. melanocephalum* has become one of the most dominant ant species in various urban habitats in southern China and negatively affected plants by attending hemipteran pests [14,15,16,17].

We hypothesize that this newly discovered tape-paving behavior can be used to monitor *S. invicta* and *T. melanocephalum* in urban areas. However, there are two premises to sustain this hypothesis: (1) tape-paving behavior should be specifically identifiable as being done by *S. invicta* or *T. melanocephalum*, meaning that only limited ant species can perform the tape-paving behavior and they can be easily distinguished from *S. invicta* and *T. melanocephalum*; and (2) the new monitoring method should be as effective as or better than traditional methods (i.e., baiting and pitfall trapping) to detect *S. invicta* and *T. melanocephalum*.

Here, we test the two premises detailed above and compare the efficacy of the taping method with traditional baits and pitfall traps to test whether this new method can be used to detect *S. invicta* and *T. melanocephalum* in urban areas. In addition, we provide the method to distinguish ant species showing the tape-paving behavior based on their morphological characteristics.

## 2. Materials and Methods

### 2.1. Efficiency of Adhesive Tapes to Detect Solenopsis invicta and Tapinoma melanocephalum

**Figure 1:**
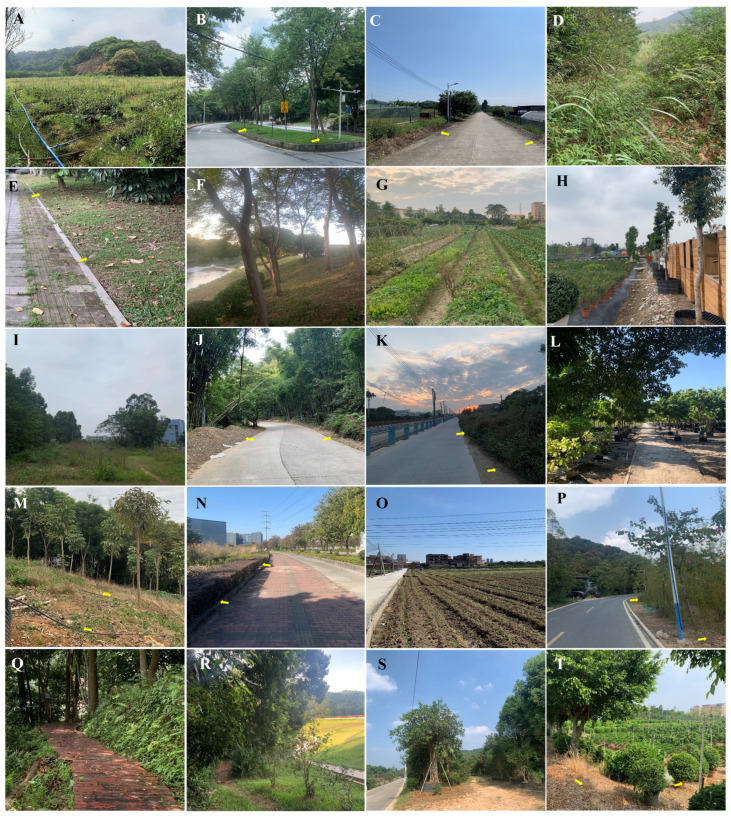
Picture of each experimental location. The yellow arrow indicates the site where bait, tape, or pitfall trap was set. (**A**–**T**) indicate location 1–20, respectively.

**Figure 2:**
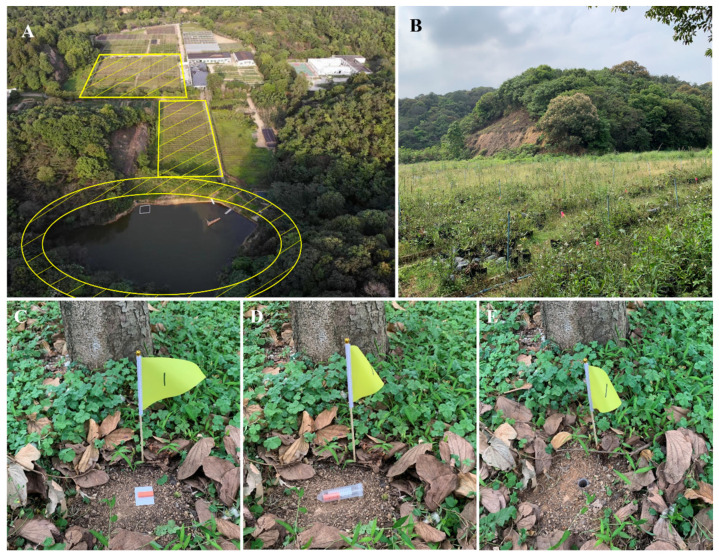
Aerial (**A**) and ground (**B**) views of experimental location one. The adhesive tapes (**C**), baiting tubes (**D**), or pitfall traps (**E**) were placed in each site of the experimental location. The hatched area shows the area where the experiment was conducted.

This experiment aimed to: (1) compare the efficiency of three types of monitors (adhesive tapes, baits, and pitfall traps) in detecting the activities of *S. invicta* and *T. melanocephalum*; and (2) determine whether other ant species perform paving behavior. The experiment was conducted between 21 April 2021 and 27 September 2022. Twenty locations representing typical urban habitats (i.e., city parks, roadsides and green belts along the road, nurseries, bushes, agricultural land, grassland, and forestry land) were selected in seven districts of Guangzhou, Guangdong, China (Figure 1; Supplementary Material: Table S1). In each location, 181–224 testing sites (a total of 3998) were randomly selected and marked, with each site being >3 m apart (Figure 2A,B). Before the experiment, weeds and litter were removed so the ant monitors could be placed on the bare soil. At each site, the following ant-monitoring methods were set:

(1) Tapes: The method provided by Wen et al. [9] was modified to set adhesive tapes. In brief, a double-sided adhesive tape (Guoxin, Dongguan, China) was pasted on one side of graph paper (50 × 50 mm) covered by a plastic membrane. Here, we pasted the tape on graph paper instead of pasting it directly on the ground because it allowed easy and rapid collection of the tape without disrupting the ants on the tape. To attract ants to approach the adhesive tape, a piece of sausage (40 × 10 × 10 mm [length × width × height], Shuanghui, Qingyuan, China) was placed on the adhesive tape, with its long axis parallel to the side of the tape. The end of the sausage was flush with one side of the tape (Figure 2C). The tape was then placed on the ground with the adhesive surface facing up. After 3 h, a high-resolution picture of the tape was taken, and the paving behavior of ants was determined if ≥10 particles were found on the tape. In addition, any tapes and sausages with ant presence (i.e., ≥1 ant was found on the surface of the tape and/or sausage) were then transferred to a sealed bag and stored in a −20 °C fridge (BCD-539WT, Haier, Qingdao, China).

(2) Baits: The method provided by Xu et al. [18] was modified. In brief, a piece of sausage (40 × 10 × 10 mm) was placed on the bottom of a 50 mL centrifuge tube (28.5 mm in diameter and 11.6 mm in length, LABSELECT^®^, Beijing, China), which was then placed flat on the ground, with the long axis on the ground (Figure 2D). Ant presence (i.e., ≥1 ant was found inside the tube) was checked after 3 h. All tubes containing ants were sealed and brought to the laboratory and stored in a −20 °C fridge.

(3) Pitfall traps: The method described by Wilder et al. [19] was modified. In brief, 20 mL of 70% propylene glycol was added into a 50 mL centrifuge tube. A hole sized to accommodate the tube (depth = 11.6 cm) was dug. The centrifuge tube was placed into the hole, such that the opening of the tube and the ground were flush. The soil was then filled into the gap between the tube and hole (Figure 2E). Ant presence (i.e., ≥1 ant was found inside the tube) was checked after 3 h, and all tubes with ants were brought to the laboratory and stored in a −20 °C fridge.

There were 1–3-day intervals between the test of each of the three methods in each location (Table S1). Because we could not dig holes in many places (e.g., parks and roadsides), pitfall traps were only tested in 2 locations (Table S1). However, both adhesive tapes and baits were used in all 20 locations. Baits was tested prior to the tapes in locations 1–11, and the reverse order was carried out in locations 12–20 to avoid any bias caused by the order. Because the setting of pitfall traps modified the surface of the ground, traps were only tested after baits and tapes.

Collected ants were identified by Dr. Zhilin Chen (Guangxi Normal University). For each ant species paving the adhesive tape, three pictures (front view, side view, and vertical view) were taken for worker and soldier ants using an ultra-depth three-dimensional microscope (VHX-500, Keyence Corporation, Osaka, Japan). In addition, three sites where each tape-paving species were found to be present were randomly selected. From samples collected at those sites, 40 workers or soldiers were randomly selected and defrosted (all workers or soldiers were defrosted if <40 individuals were collected from that site). The head of each ant was cut and placed on the graph paper facing downwards, and the head capsule width was measured using the software ImageJ (NIH Image, Bethesda, MD, USA).

### 2.2. Correlation between Head Width and Particle Area of Active Tape-Paving Ant Species

Our study showed that nine ant species paved the adhesive tapes, and five of them—*S. invicta, T. melanocephalum, Carebara affinis* (Jerdon) (Formicidae: Myrmicinae), *Pheidole nodus* Smith (Formicidae: Myrmicinae), and *Pheidole sinica* Wu & Wang (Formicidae: Myrmicinae)—are active tape-paving ant species (see results). Here, we compared their paving patterns by examining the head capsule widths of these active tape-paving species and the area of particles they transported. One species, *C. affinis*, was relatively rare and was not able to be meaningfully tested. The experiment was conducted in four locations (*S. invicta*: 23.169° N, 113.366° E; *T. melanocephalum*: 23.239° N, 113.633° E; *P. nodus*: 23.198° N, 113.299° E; *P. sinica*: 23.622° N, 113.787° E) from 15 to 26 September 2022.

The adhesive tape was set up using the same method mentioned above, but the adhesive tape and the sausage were fixed on the ground using an insect pin to avoid moving the tape during ant and particle collection. When the individual ant carrying the particle moved onto the adhesive tape, forceps were used to transfer the ant and the particle into a 2 mL Eppendorf tube (only one ant and one particle were stored in each tube). Three adhesive tapes were used for each ant species, and 40–50 ant individuals and particles they carried were collected from each adhesive tape between 10 and 90 min into the experiment. The ants were killed in a −20 °C fridge overnight, and the head capsule width of each ant was measured as mentioned above. In addition, the particle carried by each ant was placed on graph paper, and the area of the particle was measured using the software ImageJ.

### 2.3. Data Analyses

The percentage of each monitor method (tapes, baits, or pitfall traps) with *S. invicta*, *T. melanocephalum*, or other ants (non-target ants), was calculated in each of the 20 locations. We observed that many ant species could be found on the sausage placed on the adhesive tape, but only a few transported particles to pave the tape. Therefore, we also calculated the percentage of sausage with ant presence and the percentage of tapes paved by the three categories of ants (*S. invicta*, *T. melanocephalum*, or non-target ants). The percentage data were compared using the McNemar test (R code was provided by Pembury-Smith and Ruxton [20]). Here, we used the mid-*P* because Pembury-Smith and Ruxton [20] suggested that this variant can “always be used by researchers as their preferred option”. Because the data were not normally distributed, Kruskal–Wallis tests were used to compared the head width of workers or soldiers among the nine tape-paving ant species, and the Dwass–Steel–Critchlow–Fligner test (DSCF) was used for pairwise comparisons (SAS 9.4, SAS Institute, Cary, NC, USA). Head width was also compared between workers and soldiers for each species using a Kruskal–Wallis test. To compare the paving behavior of four ant species, we built a linear regression model to check if there was an interaction effect between ant head width and ant species on particle size. Because the interaction effect was significant (Supplementary Material: Table S2), we then used linear regression to investigate the correlation between head width and particle area for each ant species (data were log transformed before fitting the model).

## 3. Results

### 3.1. Efficiency of Adhesive Tapes to Detect Solenopsis invicta and Tapinoma melanocephalum

In most locations, *S. invicta* was detected in similar percentages at baits and adhesive tapes (determined by the presence of ants on the sausage and occurrence of tape paving) (Table 1, statistical results are shown in Supplementary Material: Table S3). A significantly higher percentage of adhesive tapes detected *S. invicta* than baits in locations 3, 6, and 16. However, significantly fewer adhesive tapes (determined by tape paving) detected *S. invicta* than baits in locations 11 and 15. The percentage of pitfall traps detecting *S. invicta* was similar to baits and adhesive tapes in location 13, but was lower than adhesive tapes in location 3.

In addition, there is no significant difference in detecting *T. melanocephalum* among different methods in 18 of 20 locations (Table 2, statistical results are shown in Supplementary Material: Table S4). Only in two locations did significantly fewer adhesive tapes (determined by ant infestation on the sausage and tape paving) detect *T. melanocephalum* than baits.

In almost all locations, much fewer adhesive tapes (determined by tape paving) detected non-target ants than baits and pitfall traps (Table 3, statistical results are shown in Supplementary Material: Table S5). The three methods of baits, pitfall traps, and tapes (determined by tape paving) detected 16, 10, and 7 non-target ant species, respectively (Table 4). Besides *S. invicta* and *T. melanocephalum*, seven non-target ants—*C. affinis*, *P. nodus*, *P. sinica*, *Pheidole parva* Mayr (Formicidae: Myrmicinae), *Pheidole yeensis* Forel (Formicidae: Myrmicinae), *Camponotus nicobarensis* Mayr (Formicidae: Formicinae), and *Odontoponera transversa* (Smith) (Formicidae: Ponerinae)—also paved the adhesive tape.

### 3.2. Distinguishment of Solenopsis invicta, Tapinoma melanocephalum, and Non-Target Ants Performing the Tape-Paving Behavior

*S. invicta* and *T. melanocephalum* can be easily distinguished from each other, and from other non-target ants showing tape-paving behavior. Morphologically, *S. invicta* is polymorphic (ranging from 2 to 6 mm) and individuals have a dark brown-black abdomen (Supplementary Materials: Figure S1), whereas *T. melanocephalum* is monomorphic, small, and has a yellow-colored abdomen (Supplementary Materials: Figure S2). Both *S. invicta* and *T. melanocephalum* do not have soldiers, and therefore can be easily distinguished from *P. parva*, *P. yeensis*, *P. nodus*, *P. sinica*, and *C. affinis*, which have soldiers with much a larger body size and inflated heads than workers (Table 5; Supplementary Materials: Figures S3–S7). In addition, *O. transversa* (Supplementary Materials: Figure S8) and *C. nicobarensi* (Supplementary Materials: Figure S9) have much larger body sizes than other ants. A key to ant species showed tape-paving behavior was provided (Supplementary Materials: Figure S10).

**Figure 3:**
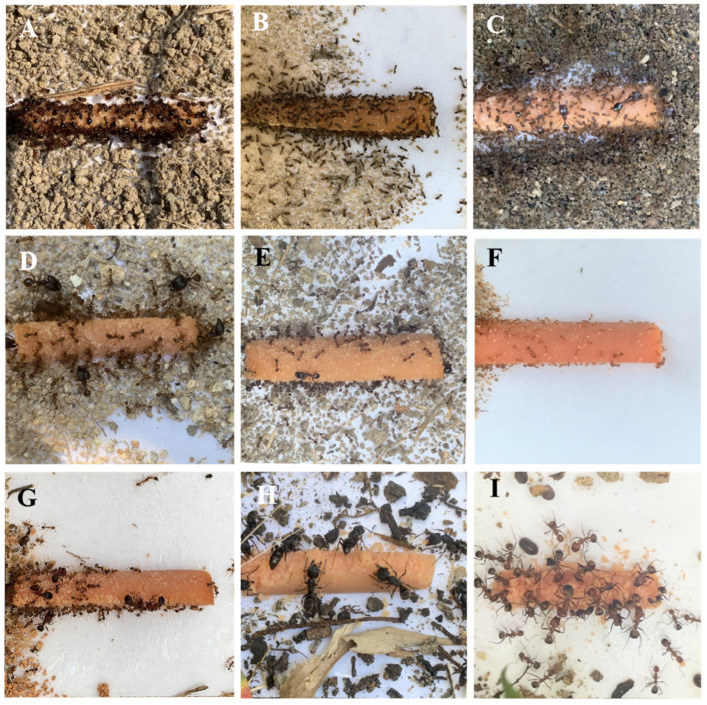
Example of adhesive tapes paved by *Solenopsis invicta* (**A**); *Tapinoma melanocephalum* (**B**); *Carebara affinis* (**C**); *Pheidole sinica* (**D**); *Pheidole nodus* (**E**); *Pheidole parva* (**F**); *Pheidole yeensis* (**G**); *Odontoponera transversa* (**H**); and *Camponotus nicobarensis* (**I**).

Furthermore, these ants can be divided into active and non-active tape-paving species. We defined *S. invicta*, *T. melanocephalum*, *C. affinis*, *P. sinica*, and *P. nodus* as the active tape-paving species because: (1) when these ants were attracted by the sausage on the tape, >50% of tapes were paved by particles (Table 4); and (2) these ants usually covered the whole tape with particles (Figure 3). In contrast, we defined *P. parva*, *P. yeensis*, *O. transversa*, and *C. nicobarensis* as non-active tape-paving species because only a small portion (<50%) of them displayed tape-paving behavior (Table 4), and they usually paved only a small part of the tape (Figure 3).

### 3.3. Correlation between Head Width and Particle Area of Active Tape-Paving Ant Species

**Figure 4:**
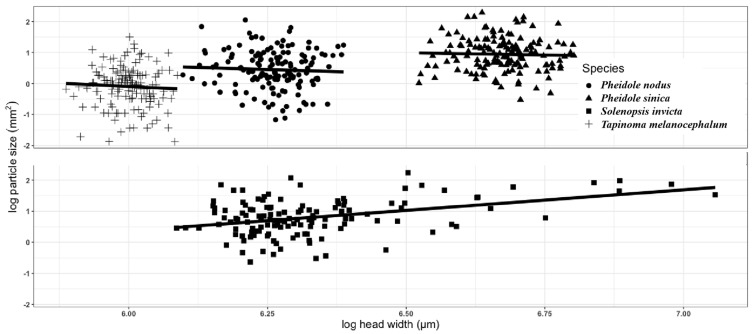
Correlation between head width and the particle size for *Solenopsis invicta*, *Pheidole sinica*, *Pheidole nodus*, or *Tapinoma melanocephalum*. The black line indicates correlation line.

For *P. nodus* and *P. sinica*, only workers engaged in particle transport and tape paving. A significant correlation between head width and the particle size was found for *S. invicta*, but was not found for *P. sinica*, *P. nodus*, and *T. melanocephalum* (Figure 4; Table 6).

## 4. Discussion

In most locations, similar or even higher percentages of adhesive tapes (determined by tape-paving) successfully detected *S. invicta* compared with baits. Only in two locations (i.e., no. 11 and 15) was *S. invicta* detected in significantly lower percentages of adhesive tapes than baits. However, it is worth noting that 109 and 100 baits and adhesive tapes detected *S. invicta* in location 11, and 62 and 52 baits and adhesive tapes detected *S. invicta* in location 15, respectively. Even though adhesive tapes and baits show differences in fire ant detecting in these two locations, the adhesive tapes still reported a sufficient number of *S. invicta* infestations. Similar results were found for *T. melanocephalum*. Interestingly, the results of bait and adhesive tape were similar in locations where <10 sites detected *S. invicta* or *T. melanocephalum*. Therefore, we believe that adhesive tapes and baits are equally effective in monitoring *S. invicta* and *T. melanocephalum*, even at the early stage of invasion when the ant number is low. Our previous studies showed that *S. invicta* also paves adhesive tapes without food (sausage) [9]. However, they transported significantly more particles when the food was available. Therefore, here we placed a sausage to enhance the tape-paving behavior. When ants cannot find particles to pave the tape (e.g., on vertical surfaces, such as tree trunks), the sausages can be attached to the tape to attract ants, which may represent an alternative baiting method to detect pest ants including *S. invicta* and *T. melanocephalum.*

Since tapes detected much fewer non-target ant species than baits in all locations (Table 3), less effort will be needed to confirm the activities of *S. invicta* and *T. melanocephalum* using adhesive tape monitors. In addition, non-target ant species with tape-paving behaviors can be rapidly distinguished using methods developed in this study. Our study also showed that pitfall traps are not quite feasible for monitoring pest ants in urban areas because digging holes in cement or hardened floors is impossible. Furthermore, it may not be allowed to set pitfall traps in city parks, green belts, and protected areas because digging the soil may negatively affect urban landscapes. In addition, unlike baits or adhesive tapes, a single pitfall trap can capture multiple ant species, complicating species identification and pest ant confirmation.

Cities are gateways for exotic ant species [21]. Many studies reported the invasion of *S. invicta* and *T. melanocephalum* in various urban habitats. For example, Chan and Guénard [22] reported that *S. invicta* invaded half of the urban agroecosystems in Hong Kong in the past 15 years, causing a 10–80% reduction in crop production and leading to significant public health issues. Qin et al. [5] reported that high abundance of *S. invicta* can be found on street trees in Guangzhou. These ants show high efficiency in transporting food items on the vertical surfaces of tree trunks, indicating their adaption to living in urban environments [5]. Our present study also showed that *S. invicta* heavily infested diverse urban habitats, such as nurseries, roadsides, green spaces, campuses, and agricultural and forest lands (especially forest edges) in Guangzhou (Table 1). We did not find *S. invicta* in only two of the locations where fire ant control was frequently carried out to prevent *S. invicta* stings for students and tourists. Overall, *S. invicta* was the most dominant ant species in 20 tested locations, which was detected by 45.6% and 46.4% of baits and adhesive tapes (determined by tape-paving), respectively (Table 4). We suggest that government and city managers pay more attention to *S. invicta* invasion in urban areas.

Beck [23] defined the tool-use behaviors of animals as the use of an object “to alter the form, position, or condition of another object, another organism, or the user itself when the user holds or carries the tool during or just prior to use”. A well-known tool-use behavior in ants is throwing particles into the liquid food or building siphon tubes to cope with water tension and facilitate liquid feeding [24,25,26]. Ants also use particles to absorb liquid food and transport soaked particles back to the nest [27,28,29]. However, these behaviors were only observed in the ant subfamily myrmicinae. Lőrinczi et al. [28] stated that myrmicinae ant species do not have greatly distensible crops that can carry large amount of liquid food, and therefore they have evolved such behaviors to handle and consume liquid food. Our previous study showed that tape-paving behavior is another type of tool used by *S. invicta* [9]. The present study expands the record of this behavior to diverse ant taxa, including myrmicinae, dolichoderinae, formicinae, and ponerinae species.

Previous research about the correlation between ant body and particle size (CBPS) mainly focused on foragers and the food (baits and seeds) that ants collected or preyed upon [30,31,32,33]. To our best knowledge, this study was the first one that quantitatively investigated the link between the size of ant and non-food particles they transport. A larger particle would cover more viscose surface. Thus, workers would choose the particles with the maximum size they can carry. If there were sufficient particles of various size, a positive CBPS would be expected [34]. In our study, the positive CBPS was only detected in *S. invicta*, which is probably because the fire ant is the only polymorphic species that has a wide range of body size of workers, and the head capsule size (maximum mandible width) may dictate the size of particle that can be carried. This positive correlation is consistent with previous studies which investigated the bait size selection by *S. invicta* [35,36]. It is worth noting that comparing this study with previous studies that addressed food particles may not be appropriate because the purpose of transporting food and soil is different.

One limitation of this study is that we only set experiments in urban areas of Guangzhou. It would be essential to test the efficiency of taping to monitor *S. invicta* and *T. melanocephalum* in other habitat types and invaded regions (countries). It is also important to conduct field studies on the broader region to investigate the tape-paving behavior of non-target ant species and determine whether they can be easily distinguished from *S. invicta* and *T. melanocephalum*.

## 5. Conclusions

Our study showed that: (i) adhesive tapes are as effective as baits and pitfall traps in monitoring *S. invicta* and *T. melanocephalum* in urban areas in southern China. In addition, adhesive tapes detected a lower proportion and fewer species of non-target ants than baits and pitfall traps; (ii) tape-paving behaviors were observed in different ant taxa. Although some non-target ants also showed tape-paving behaviors, they can be easily distinguished from *S. invicta* and *T. melanocephalum*; (iii) the correlation between head width and the size of particles transported was significant in *S. invicta*, but was not significant in other active tape-paving species (i.e., *T. melanocephalum*, *P. nodus*, and *P. sinica*).

## Figures and Tables

**Table 1:** Number of monitors (bait, tape, or pitfall trap) detecting *Solenopsis invicta* at each location. “-” indicates pitfall traps were not tested in the location. Different letters within the same row indicate significant differences (*p* < 0.05). Statistical results are shown in Supplementary Material: Table S3.

No.	No. of Sites	Number of Sites Detecting *Solenopsis invicta*			
		Baiting	Taping		Pitfall Trapping
			Determined by Ant Infestation on the Sausage	Determined by Tape Paving	
1	224	211 a	215 a	214 a	-
2	224	0 a	0 a	0 a	-
3	199	74 b	107 a	106 a	86 b
4	200	181 a	184 a	184 a	-
5	199	190 b	197 a	193 ab	-
6	197	73 b	86 a	86 a	-
7	200	145 ab	154 a	138 b	-
8	200	134 a	143 a	141 a	-
9	199	134 a	131 a	129 a	-
10	200	50 a	46 a	44 a	-
11	199	109 a	100 b	100 b	-
12	200	60 a	58 a	54 a	-
13	200	69 a	67 a	65 a	70 a
14	200	75 a	74 a	72 a	-
15	195	62 a	60 a	52 b	-
16	198	66 b	78 a	77 a	-
17	196	0 a	0 a	0 a	-
18	196	2 a	2 a	2 a	-
19	191	93 a	99 a	99 a	-
20	181	95 a	99 a	97 a	-

**Table 2:** Number of monitors (bait, tape, or pitfall trap) detecting *Tapinoma melanocephalum* at each location. “-” indicates pitfall traps were not tested in the location. Different letters within the same row indicate significant differences (*p* < 0.05). Statistical results are shown in Supplementary Material: Table S4.

No.	No. of Sites	Number of Sites Detecting *Tapinoma melanocephalum*			
		Baiting	Taping		Pitfall Trapping
			Determined by Ant Infestation on the Sausage	Determined by Tape Paving	
1	224	6 a	2 a	2 a	-
2	224	92 a	82 a	82 a	-
3	199	98 a	85 b	85 b	97 a
4	200	0 a	0 a	0 a	-
5	199	0 a	0 a	0 a	-
6	197	19 a	18 a	18 a	-
7	200	2 a	2 a	2 a	-
8	200	14 a	16 a	16 a	-
9	199	19 a	17 a	17 a	-
10	200	0 a	0 a	0 a	-
11	199	33 a	38 a	37 a	-
12	200	2 a	1 a	1 a	-
13	200	8 a	6 a	6 a	5 a
14	200	17 a	15 a	15 a	-
15	195	7 a	7 a	7 a	-
16	198	17 a	6 b	5 b	-
17	196	0 a	0 a	0 a	-
18	196	9 a	9 a	9 a	-
19	191	2 a	2 a	2 a	-
20	181	5 a	5 a	4 a	-

**Table 3:** Number of monitors (bait, tape, or pitfall trap) detecting non-target ants (ants other than *Solenopsis invicta* and *Tapinoma melanocephalum*) in each location. “-” indicates pitfall traps were not tested in the location. Different letters within the same row indicate significant differences (*p* < 0.05). Statistical results are shown in Supplementary Material: Table S5.

No.	No. of Sites	Number of Sites Detecting Non-Target Ants			
		Baiting	Taping		Pitfall Trapping
			Determined by Ant Infestation on the Sausage	Determined by Tape Paving	
1	224	4 ab	6 a	0 b	-
2	224	124 a	120 a	5 b	-
3	199	9 b	5 bc	1 c	24 a
4	200	16 a	14 a	1 b	-
5	199	2 a	2 a	0 a	-
6	197	52 b	64 a	4 c	-
7	200	2 a	1 a	0 a	-
8	200	4 ab	8 a	0 b	-
9	199	7 a	11 a	0 b	-
10	200	114 a	124 a	2 b	-
11	199	24 a	31 a	0 b	-
12	200	43 a	41 a	0 b	-
13	200	95 a	104 a	5 b	99 a
14	200	45 b	53 a	0 c	-
15	195	78 a	80 a	0 b	-
16	198	47 b	70 a	3 c	-
17	196	182 a	175 a	148 b	-
18	196	120 b	130 a	74 c	-
19	191	19 a	17 a	0 b	-
20	181	51 a	45 a	5 b	-

**Table 4:** Species and percentage of each ant species detected by baits, adhesive tapes (either determined by ant infestation on the sausage or tape paving), or pitfall traps. In total, 3998, 3998, and 399 of baits, adhesive tapes, and pitfall traps were tested, respectively. “*” after the species name indicates that an ant species performed tape-paving behavior.

Subfamily	Species	Baiting		Taping (Determined by Ant Infestation on the Sausage)		Taping (Determined by Tape Paving)		Pitfall Trapping	
		No. of Baits Detecting the Ant Species	Percentage of Baits Detecting the Ant Species (%)	No. of Tapes Detecting the Ant Species	Percentage of Tapes Detecting the Ant Species (%)	No. of Tapes Detecting the Ant Species	Percentage of Tapes Detecting the Ant Species (%)	No. of Pitfall Traps Detecting the Ant Species	Percentage of Pitfall Traps Detecting the Ant Species (%)
Myrmicinae	*Solenopsis invicta* *	1823	45.60	1900	47.52	1853	46.35	156	39.10
Dolichoderinae	*Tapinoma melanocephalum* *	350	8.75	311	7.78	308	7.70	102	25.56
Myrmicinae	*Pheidole sinica* *	135	3.38	126	3.15	118	2.95	0	0
Ponerinae	*Odontoponera transversa* *	134	3.35	145	3.63	37	0.93	28	7.02
Formicinae	*Paratrechina longicornis*	133	3.33	104	2.60	0	0	7	1.75
Myrmicinae	*Pheidole parva* *	130	3.25	138	3.45	3	0.08	12	3.01
Myrmicinae	*Monomorium chinense*	104	2.60	108	2.70	0	0	21	5.26
Myrmicinae	*Pheidole nodus* *	87	2.18	95	2.38	58	1.45	7	1.75
Formicinae	*Camponotus nicobarensis* *	80	2.00	98	2.45	7	0.18	16	4.01
Myrmicinae	*Tetramorium caespitum*	48	1.20	58	1.45	0	0	7	1.75
Myrmicinae	*Crematogaster rogenhoferi*	57	1.43	64	1.60	0	0	4	1.00
Ponerinae	*Diacamma rugosum*	31	0.78	36	0.90	0	0	9	2.26
Myrmicinae	*Monomorium pharaonis*	25	0.63	21	0.53	0	0	0	0
Myrmicinae	*Pheidole pieli*	20	0.50	18	0.45	0	0	0	0
Formicinae	*Anoplolepis gracilipes*	18	0.50	34	0.94	0	0	0	0
Formicinae	*Polyrhachis dives*	15	0.41	18	0.50	0	0	0	0
Myrmicinae	*Carebara affinis* *	14	0.39	14	0.39	14	0.39	0	0
Myrmicinae	*Pheidole yeensis* *	10	0.28	9	0.25	4	0.11	12	3.01

**Table 5:** Head width (mean ± SE) of nine ants showing tape-paving behavior. Different letters within the same column indicate significant differences among different ant species (*p* < 0.05). “*” indicates significant differences between worker and soldiers for each ant species (*p* < 0.05). “-” indicates no soldier caste was presented for this species.

Tape-Paving Ant Species	Head Width of Workers (mm)	Head Width of Soldiers (mm)	χ^2^	df	*p*-Value
*Solenopsis invicta*	0.583 ± 0.150 d	-	-	-	-
*Tapinoma melanocephalum*	0.402 ± 0.019 g	-	-	-	-
*Pheidole parva*	0.450 ± 0.020 f	0.783 ± 0.050 d	106.78	1	<0.0001 *
*Pheidole yeensis*	0.597 ± 0.021 d	1.720 ± 0.057 c	91.14	1	<0.0001 *
*Pheidole nodus*	0.536 ± 0.028 e	1.717 ± 0.041 c	113.98	1	<0.0001 *
*Pheidole sinica*	0.799± 0.038 c	2.971 ± 0.038 a	57.51	1	<0.0001 *
*Carebara affinis*	0.536 ± 0.027 e	1.888 ± 0.185 b	105.28	1	<0.0001 *
*Odontoponera transversa*	1.966 ± 0.049 a	-	-	-	-
*Camponotus nicobarensis*	1.896 ± 0.035 b	-	-	-	-
χ^2^	830.44	159.93			
df	8	4			
*p*-value	<0.0001	<0.0001			

**Table 6:** Results of linear regression models with particle size as dependent variable and ant head width as independent variable for each species.

Species		Estimate	SD	*t*-Value	*p*-Value
*Solenopsis invicta*	Intercept	−7.583	1.746	−4.344	<0.001
	Head width	1.325	0.276	4.807	<0.001
*Pheidole sinica*	Intercept	2.861	4.956	0.577	0.565
	Head width	−0.287	0.743	−0.387	0.699
*Pheidole nodus*	Intercept	3.786	5.735	0.660	0.510
	Head width	−0.534	0.916	−0.583	0.561
*Tapinoma melanocephalum*	Intercept	5.195	7.983	0.651	0.516
	Head width	−0.882	1.331	−0.663	0.509

## Data Availability

The raw data and materials will be made available by the authors, without undue reservation, to any qualified researchers.

## Supplementary Materials

The following supporting information can be downloaded at: https://www.mdpi.com/article/10.3390/insects14030219/s1, Table S1: Location and basic information of field experiments; Table S2. ANOVA summary of the linear regression model with particle size as dependent variable and the interaction of head width and ant species as independent variable; Table S3. Number of monitors (bait, tape, or pitfall trap) detecting *Solenopsis invicta* at each location; Table S4. Number of monitors (bait, tape, or pitfall trap) detecting *Tapinoma melanocephalum* at each location; Table S5. Number of monitors (bait, tape, or pitfall trap) detecting non-target ants (ants other than *Solenopsis invicta* and *Tapinoma melanocephalum*) in each location; Figure S1. Front view, side view, and vertical view of the red imported fire ant, *Solenopsis invicta* Buren (Formicidae: Myrmicinae); Figure S2. Front view, side view, and vertical view of the ghost ant, *Tapinoma melanocephalum* (Fabricius) (Formicidae: Dolichoderinae); Figure S3. Front view, side view, and vertical view of *Pheidole parva* Mayr (Formicidae: Myrmicinae); Figure S4. Front view, side view, and vertical view of *Pheidole yeensis* Forel (Formicidae: Myrmicinae); Figure S5. Front view, side view, and vertical view of *Pheidole nodus* Smith (Formicidae: Myrmicinae); Figure S6. Front view, side view, and vertical view of *Pheidole sinica* Wu & Wang (Formicidae: Myrmicinae); Figure S7. Front view, side view, and vertical view of *Carebara affinis* (Jerdon) (Formicidae: Myrmicinae); Figure S8. Front view, side view, and vertical view of *Odontoponera transversa* (Smith) (Formicidae: Ponerinae); Figure S9. Front view, side view, and vertical view of *Camponotus nicobarensis* Mayr (Formicidae: Formicinae); Figure S10. A key of tape-paving ant species (*Solenopsis invicta*, *Tapinoma melanocephalum*, *Pheidole* spp., *Carebara affinis*, *Camponotus nicobarensis*, and *Odontoponera transversa*) in this study.
