# Supplementary material for: Paving Behavior in Ants and Its Potential Application in Monitoring Two Urban Pest Ants, Solenopsis invicta and Tapinoma melanocephalum
Source: Insects. 2023 Feb 23;14(3):219. doi: 10.3390/insects14030219 (PMC10054563; doi:10.3390/insects14030219)
Supplement: Supplementary file 1 [file insects-14-00219-s001.zip › insects-2199293-SI.pdf]

Paving Behavior in Ants and its Potential Application in Monitoring Two Urban Pest Ants, *Solenopsis invicta* and *Tapinoma melanocephalum*

Supplementary Materials

Liming Shen [1,†], Chao Wen [2,†], Xuan Chen [3,†], Yan Hua [4], Chengju Du [1], Jiacheng Cai [5], Xiujun Wen [1], Lei Wang [6,*] and Cai Wang [1,*]

[1] College of Forestry and Landscape Architecture, South China Agricultural University, Guangzhou 510642, China; shenliming@stu.scau.edu.cn (L.S.); duchengju@stu.scau.edu.cn (C.D.); wenxiujun@scau.edu.cn (X.W.)
[2] School of Grassland Science, Beijing Forestry University, Beijing 100083, China; wenchao@bjfu.edu.cn
[3] Department of Biological Sciences, Salisbury University, Salisbury, MD 21801, USA; xxchen@salisbury.edu
[4] Guangdong Provincial Key Laboratory of Silviculture, Protection and Utilization, Guangdong Academy of Forestry, Guangzhou 510520, China; wildlife530@hotmail.com
[5] Department of Mathematical Sciences, Salisbury University, Salisbury, MD 21801, USA; jxcai@salisbury.edu
[6] College of Plant Protection, South China Agricultural University, Guangzhou 510642, China
* Correspondence: leiwang@scau.edu.cn (L.W.); wangcai@scau.edu.cn (C.W.)
† These authors contributed equally to this work.

Table S1: Location and basic information of field experiments.

No.	Location	GPS	Habitat Type	Monitoring Method	Date (yyyy/mm/dd)	Weather
1	Tianlu Lake, Huangpu District	23.252° N 113.411° E	Nursery	Baiting	2021/04/21	Cloudy
				Taping	2021/04/23	
2	Campus of South China Agricultural University (SCAU), Tianhe District	23.162° N 113.355° E	Green Space	Baiting	2021/07/22	Sunny
				Taping	2021/07/24	
3	Zengcheng Teaching and Research Bases, SCAU, Zengcheng District	23.239° N 113.633° E	Roadside and green belts along the road	Baiting	2021/10/26	Cloudy
				Taping	2021/10/28	
				Pitfall Trapping	2021/10/31	
4	Furong Travel Resort, Huadu District	23.512° N 113.234° E	Forestry Land	Baiting	2021/11/02	Sunny
				Taping	2021/11/04	
5	Qilingbei Research Station, SCAU, Tianhe District	23.169° N 113.366° E	Roadside and green belts along the road	Baiting	2021/11/11	Sunny
				Taping	2021/11/13	
6	River bank of Qilingbei Research Station, SCAU, Tianhe District	23.166° N 113.361° E	Grass Land	Baiting	2021/11/12	Sunny
				Taping	2021/11/14	
7	Songmao Nursery, Baiyun District	23.367° N 113.396° E	Agricultural land	Baiting	2021/12/09	Cloudy
				taping	2021/12/13	
8	Seedling nursery near Institute of Gardening of Guangzhou, Baiyun District	23.261° N 113.302° E	Nursery	Baiting	2021/11/18	Overcast Sky
				Taping	2021/11/21	
9	Around the Sports Ground of South China Agricultural University (SCAU), Tianhe District	23.161° N 113.369° E	Bush	Baiting	2021/11/17	Overcast Sky
				Taping	2021/11/19	
10	Shangdongkeng Village, Baiyun District	23.270° N 113.436° E	Roadside and green belts along the road	Baiting	2021/12/03	Sunny
				Taping	2021/12/12	
11	Xuhui Housing Estate, Baiyun District	23.370° N 113.387° E	Roadside and green belts along the road	Baiting	2021/12/10	Cloudy
				Taping	2021/12/15	
12		23.261° N	Nursery	Taping	2021/12/01	Sunny

	Institute of Gardening of Guangzhou (Dongping Bases), Baiyun District	113.304° E		Baiting	2021/12/06	
13	Zhongluotan Nursery, Baiyun District	23.364° N 113.440° E	Nursery	Taping	2021/12/02	Overcast Sky
				Baiting	2021/12/07	
				Pitfall Trapping	2021/12/17	
14	Yingchun Road, Huadu District	23.353° N 113.251° E	Roadside and green belts along the road	Taping	2021/11/31	Sunny
				Baiting	2021/12/04	
15	Longda Village, Baiyun District	23.392° N 113.388° E	Agricultural Land	Taping	2022/03/08	Sunny
				Baiting	2022/03/12	
16	Maofeng Mountain, Baiyun District	23.270° N 113.443° E	Roadside and Bush	Taping	2022/03/13	Sunny
				Baiting	2022/03/15	
17	Shimen National Forest Park, Conghua District	23.622° N 113.787° E	Roadside and Forestry land	Taping	2022/07/12	Sunny
				Baiting	2022/07/14	
18	Baiyun Mountain, Baiyun District	23.198° N 113.299° E	Forestry land and grass land	Taping	2022/07/24	Sunny
				Baiting	2022/07/26	
19	Wudantian, Zengcheng District	22.921° N 113.511° E	Nursery	Taping	2022/09/17	Sunny
				Baiting	2022/09/20	
20	Zhonglyu Mangrove Nursery, Panyu District	23.386° N 113.589° E	Nursery and Roadside	Taping	2022/09/25	Sunny
				Baiting	2022/09/27	

Table S2. ANOVA summary of the linear regression model with particle size as dependent variable and head width and ant species as independent variables.

	DF	Sum Sq	Mean Sq	*F*-value	*P*-value
Head width	1	81.23	81.23	227.66	<0.001
Ant species	3	15.43	5.14	14.41	<0.001
Head width × Ant species	3	3.21	1.07	3	0.03
residuals	561	200.17	0.36		

Table S3. Number of monitors (bait, tape, or pitfall trap) detecting *Solenopsis invicta* at each location. "-" indicates pitfall traps were not tested in the location. Different letters within the same row indicate significant differences ($p < 0.05$).

No.	No. of sites	Number of sites detecting *Solenopsis invicta*				Statistical Results	
		Baiting (B)	Taping: Determined by ant infestation on the sausage (SA)	Taping: Determined by tape paving (PA)	Pitfall Trapping (PT)	Comparisons	Mid-P
1	224	211 a	215 a	214 a	-	B vs SA	0.2266
						B vs PA	0.3877
						PA vs SA	0.5000
2	224	0 a	0 a	0 a	-	B vs SA	1.0000
						B vs PA	1.0000
						PA vs SA	1.0000
3	199	74 b	107 a	106 a	86 b	B vs SA	< 0.0001
						B vs PA	< 0.0001
						PA vs SA	0.5000
						B vs PT	0.0854
						SA vs PT	0.0054
						PA vs PT	0.0086
4	200	181 a	184 a	184 a	-	B vs SA	0.2186
						B vs PA	0.2186
						PA vs SA	1.000
5	199	190 b	197 a	193 ab	-	B vs SA	0.0386
						B vs PA	0.3877
						PA vs SA	0.0625
6	197	73 b	86 a	86 a	-	B vs SA	0.0161
						B vs PA	0.0161
						PA vs SA	1.000
7	200	145 ab	154 a	138 b	-	B vs SA	0.0755
						B vs PA	0.2430
						PA vs SA	< 0.0001
8	200	134 a	143 a	141 a	-	B vs SA	0.1516
						B vs PA	0.0525
						PA vs SA	0.2500
9	199	134 a	131 a	129 a	-	B vs SA	0.4240
						B vs PA	0.2101
						PA vs SA	0.2500

10	200	50 a	46 a	44 a	-	B vs SA	0.2500
						B vs PA	0.1892
						PA vs SA	0.2500
11	199	109 a	100 b	100 b	-	B vs SA	0.0414
						B vs PA	0.0414
						PA vs SA	1.0000
12	200	60 a	58 a	54 a	-	B vs SA	0.6291
						B vs PA	0.1435
						PA vs SA	0.0625
13	200	69 a	67 a	65 a	70 a	B vs SA	0.4531
						B vs PA	0.1797
						PA vs SA	0.2500
						B vs PT	0.6875
						SA vs PT	0.2891
						PA vs PT	0.1094
14	200	75 a	74 a	72 a	-	B vs SA	0.8238
						B vs PA	0.5235
						PA vs SA	0.2500
15	195	62 a	60 a	52 b	-	B vs SA	0.6636
						B vs PA	0.0347
						PA vs SA	0.0078
16	198	66 b	78 a	77 a	-	B vs SA	0.0192
						B vs PA	0.0290
						PA vs SA	1.0000
17	196	0 a	0 a	0 a	-	B vs SA	1.0000
						B vs PA	1.0000
						PA vs SA	1.0000
18	196	2 a	2 a	2 a	-	B vs SA	1.0000
						B vs PA	1.0000
						PA vs SA	1.0000
19	191	93 a	99 a	99 a	-	B vs SA	0.0654
						B vs PA	0.0654
						PA vs SA	1.0000
20	181	95 a	99 a	97 a	-	B vs SA	0.2265
						B vs PA	0.5811
						PA vs SA	0.2500

Table S4. Number of monitors (bait, tape, or pitfall trap) detecting *Tapinoma melanocephalum* at each location. "-" indicates pitfall traps were not tested in the location. Different letters within the same row indicate significant differences ($p < 0.05$).

No.	No. of sites	Number of sites detecting *Tapinoma melanocephalum*				Statistical Results	
		Baiting (B)	Taping: Determined by ant infestation on the sausage (SA)	Taping: Determined by tape paving (PA)	Pitfall Trapping (PT)	Comparisons	Mid-*P*
1	224	6 a	2 a	2 a	-	B vs SA	0.0625
						B vs PA	0.0625
						PA vs SA	1.0000
2	224	92 a	82 a	82 a	-	B vs SA	0.0576
						B vs PA	0.0576
						PA vs SA	1.0000
3	199	98 a	85 b	85 b	97 a	B vs SA	0.0385
						B vs PA	0.0385
						PA vs SA	1.0000
						B vs PT	0.8957
						SA vs PT	0.0919
						PA vs PT	0.0919
4	200	0 a	0 a	0 a	-	B vs SA	1.0000
						B vs PA	1.0000
						PA vs SA	1.0000
5	199	0 a	0 a	0 a	-	B vs SA	1.0000
						B vs PA	1.0000
						PA vs SA	1.0000
6	197	19 a	18 a	18 a	-	B vs SA	0.8036
						B vs PA	0.8036
						PA vs SA	1.0000
7	200	2 a	2 a	2 a	-	B vs SA	1.0000
						B vs PA	1.0000
						PA vs SA	1.0000
8	200	14 a	16 a	16 a	-	B vs SA	0.3750
						B vs PA	0.3750
						PA vs SA	1.0000
9	199	19 a	17 a	17 a	-	B vs SA	0.4531
						B vs PA	0.4531
						PA vs SA	1.0000

10	200	0 a	0 a	0 a	-	B vs SA	1.0000
						B vs PA	1.0000
						PA vs SA	1.0000
11	199	33 a	38 a	37 a	-	B vs SA	0.2101
						B vs PA	0.3323
						PA vs SA	0.5000
12	200	2 a	1 a	1 a	-	B vs SA	0.5000
						B vs PA	0.5000
						PA vs SA	1.0000
13	200	8 a	6 a	6 a	5 a	B vs SA	0.3750
						B vs PA	0.3750
						PA vs SA	1.0000
						B vs PT	0.4531
						SA vs PT	0.1797
						PA vs PT	0.1797
14	200	17 a	15 a	15 a	-	B vs SA	0.4231
						B vs PA	0.4231
						PA vs SA	1.0000
15	195	7 a	7 a	7 a	-	B vs SA	1.0000
						B vs PA	1.0000
						PA vs SA	1.0000
16	198	17 a	6 b	5 b	-	B vs SA	0.0005
						B vs PA	0.0002
						PA vs SA	1.0000
17	196	0 a	0 a	0 a	-	B vs SA	1.0000
						B vs PA	1.0000
						PA vs SA	1.0000
18	196	9 a	9 a	9 a	-	B vs SA	1.0000
						B vs PA	1.0000
						PA vs SA	1.0000
19	191	2 a	2 a	2 a	-	B vs SA	1.0000
						B vs PA	1.0000
						PA vs SA	1.0000
20	181	5 a	5 a	4 a	-	B vs SA	1.0000
						B vs PA	1.0000
						PA vs SA	0.5000

Table S5. Number of monitors (bait, tape, or pitfall trap) detecting non-target ants (ants other than *Solenopsis invicta* and *Tapinoma melanocephalum*) in each location. "-" indicates pitfall traps were not tested in the location. Different letters within the same row indicate significant differences (p < 0.05).

No.	No. of sites	Number of sites detecting non-target ants				Statistical Results	
			Taping				
		Baiting (B)	Determined by ant infestation on the sausage (SA)	Determined by tape paving (PA)	Pitfall Trapping (PT)	Comparisons	Mid-*P*
1	224	4 ab	6 a	0 b	-	B vs SA	0.5488
						B vs PA	0.0625
						PA vs SA	0.0156
2	224	124 a	120 a	5 b	-	B vs SA	0.5682
						B vs PA	< 0.0001
						PA vs SA	< 0.0001
3	199	9 b	5 bc	1 c	24 a	B vs SA	0.3018
						B vs PA	0.0117
						PA vs SA	0.0625
						B vs PT	0.0052
						SA vs PT	< 0.0001
						PA vs PT	< 0.0001
4	200	16 a	14 a	1 b	-	B vs SA	0.3750
						B vs PA	< 0.0001
						PA vs SA	0.0001
5	199	2 a	2 a	0 a	-	B vs SA	1.0000
						B vs PA	0.2500
						PA vs SA	0.2500
6	197	52 b	64 a	4 c	-	B vs SA	0.0294
						B vs PA	< 0.0001
						PA vs SA	< 0.0001
7	200	2 a	1 a	0 a	-	B vs SA	1.0000
						B vs PA	0.2500
						PA vs SA	0.5000
8	200	4 ab	8 a	0 b	-	B vs SA	0.1250
						B vs PA	0.0625
						PA vs SA	0.0039
9	199	7 a	11 a	0 b	-	B vs SA	0.2266
						B vs PA	0.0078
						PA vs SA	0.0005

10	200	114 a	124 a	2 b	-	B vs SA	0.0989
						B vs PA	< 0.0001
						PA vs SA	< 0.0001
11	199	24 a	31 a	0 b	-	B vs SA	0.0768
						B vs PA	< 0.0001
						PA vs SA	< 0.0001
12	200	43 a	41 a	0 b	-	B vs SA	0.6636
						B vs PA	< 0.0001
						PA vs SA	< 0.0001
13	200	95 a	104 a	5 b	99 a	B vs SA	0.0872
						B vs PA	< 0.0001
						PA vs SA	< 0.0001
						B vs PT	0.4421
						SA vs PT	0.2632
						PA vs PT	< 0.0001
14	200	45 b	53 a	0 c	-	B vs SA	0.0225
						B vs PA	< 0.0001
						PA vs SA	< 0.0001
15	195	78 a	80 a	0 b	-	B vs SA	0.7201
						B vs PA	< 0.0001
						PA vs SA	< 0.0001
16	198	47 b	70 a	3 c	-	B vs SA	0.0056
						B vs PA	< 0.0001
						PA vs SA	< 0.0001
17	196	182 a	175 a	148 b	-	B vs SA	0.0768
						B vs PA	< 0.0001
						PA vs SA	< 0.0001
18	196	120 b	130 a	74 c	-	B vs SA	0.0127
						B vs PA	< 0.0001
						PA vs SA	< 0.0001
19	191	19 a	17 a	0 b	-	B vs SA	0.2500
						B vs PA	< 0.0001
						PA vs SA	< 0.0001
20	181	51 a	45 a	5 b	-	B vs SA	0.0923
						B vs PA	< 0.0001
						PA vs SA	< 0.0001

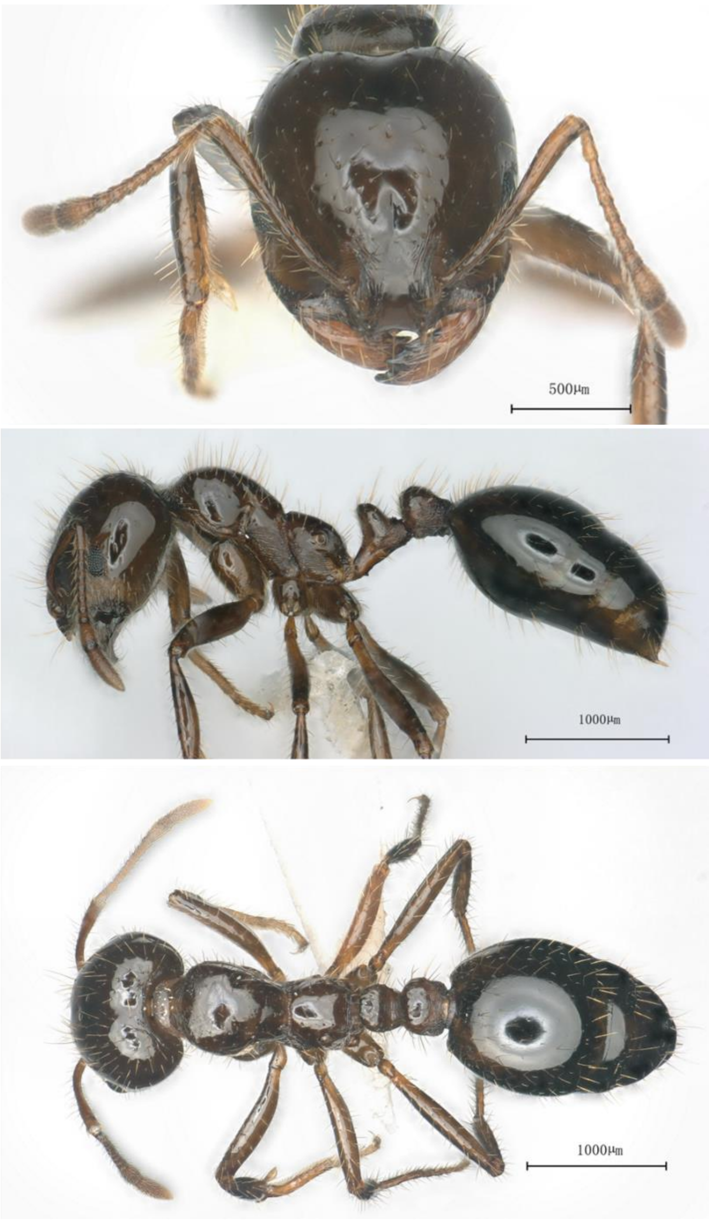

Figure S1. Front view, side view, and vertical view of the red imported fire ant, *Solenopsis invicta* Buren (Formicidae: Myrmicinae).

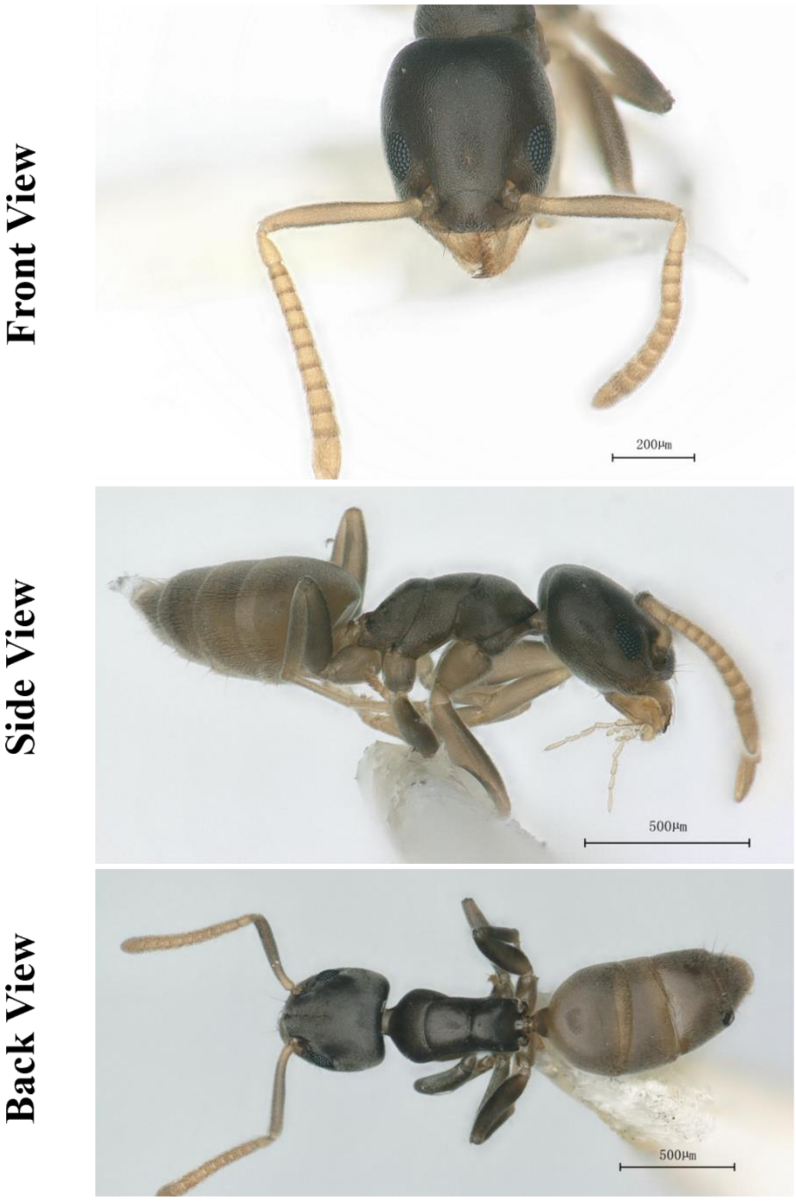

Figure S2. Front view, side view, and vertical view of the ghost ant, *Tapinoma melanocephalum* (Fabricius) (Formicidae: Dolichoderinae).

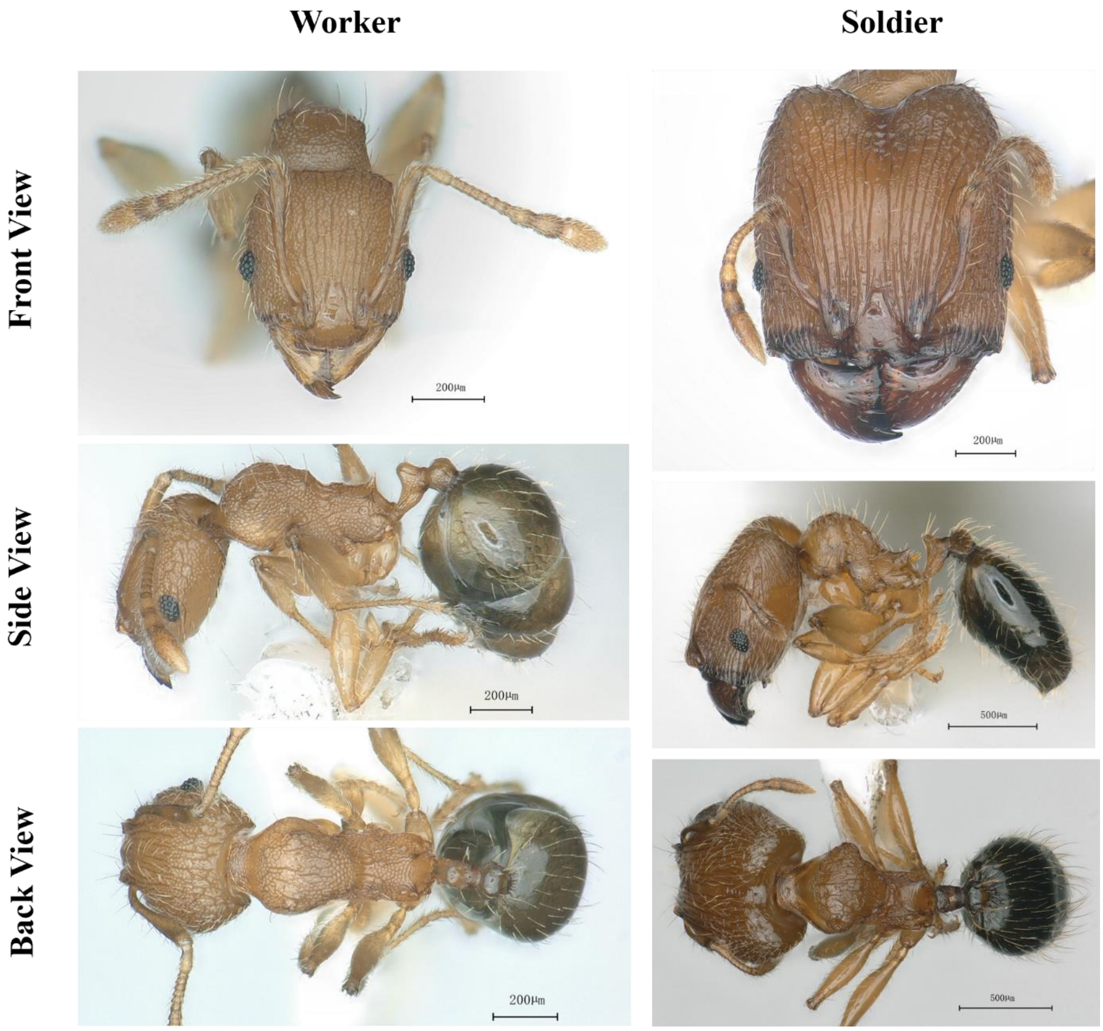

Figure S3. Front view, side view, and vertical view of *Pheidole parva* Mayr (Formicidae: Myrmicinae).

.

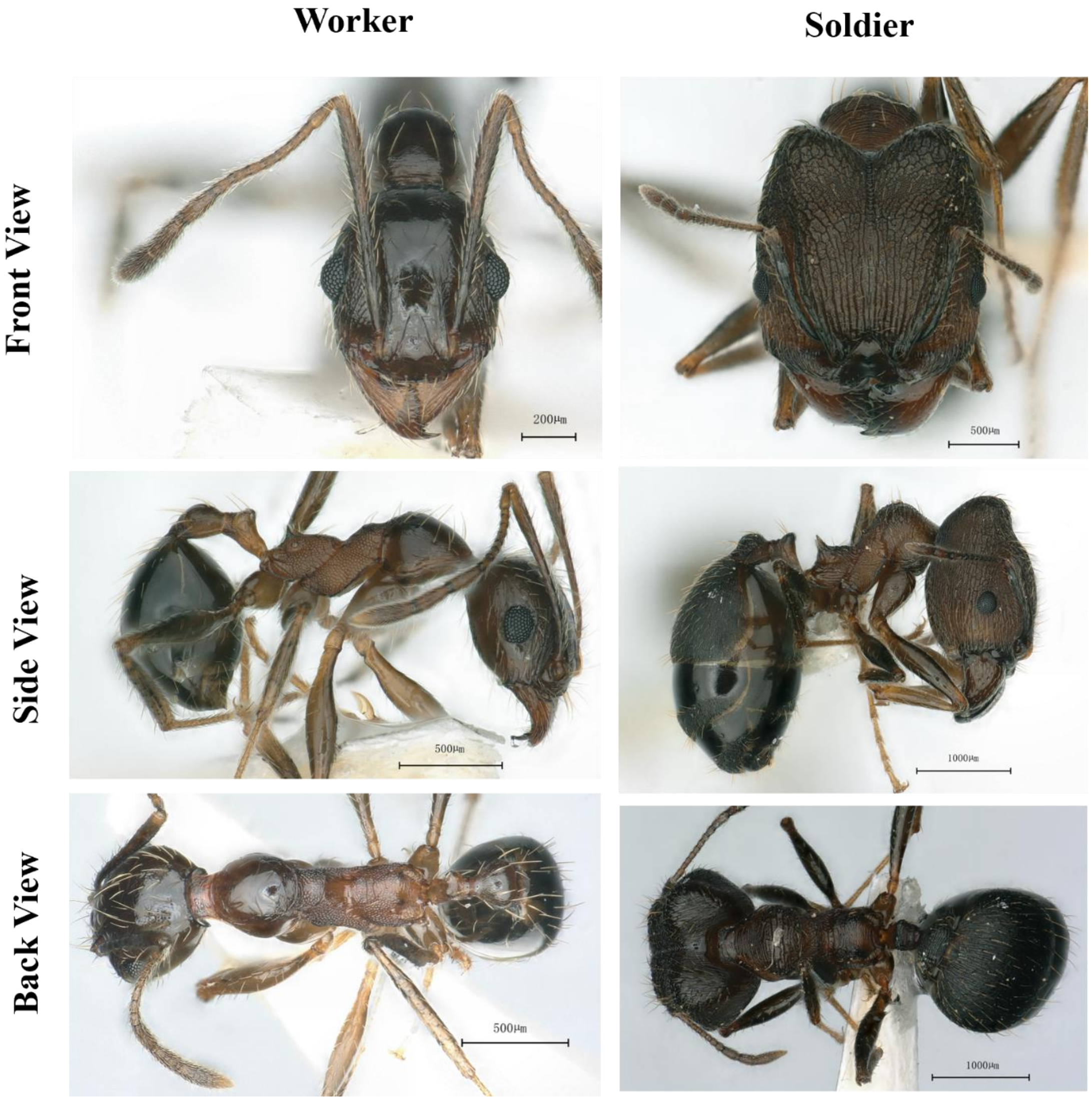

Figure S4. Front view, side view, and vertical view of *Pheidole yeensis* Forel (Formicidae: Myrmicinae).

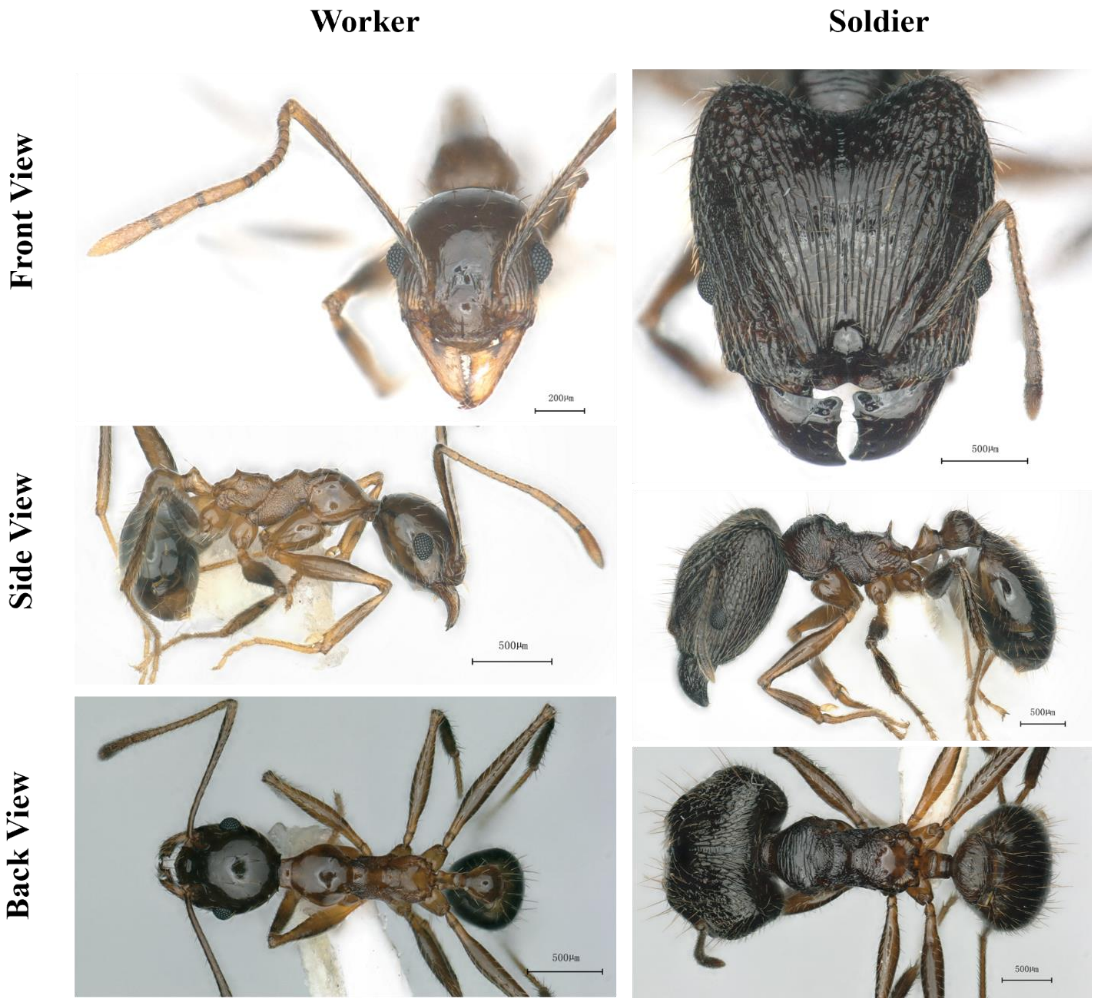

Figure S5. Front view, side view, and vertical view of *Pheidole nodus* Smith (Formicidae: Myrmicinae).

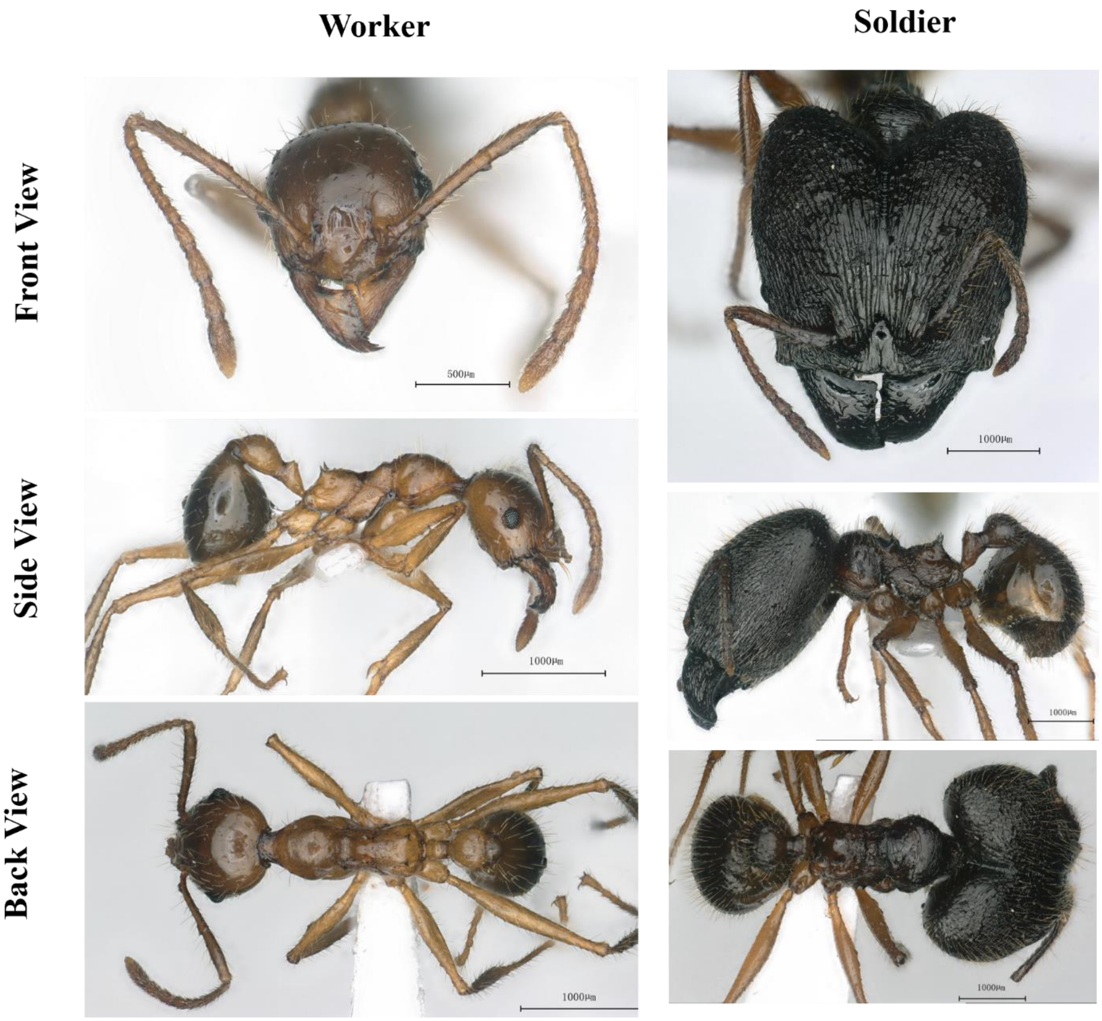

Figure S6. Front view, side view, and vertical view of *Pheidole sinica* Wu & Wang (Formicidae: Myrmicinae).

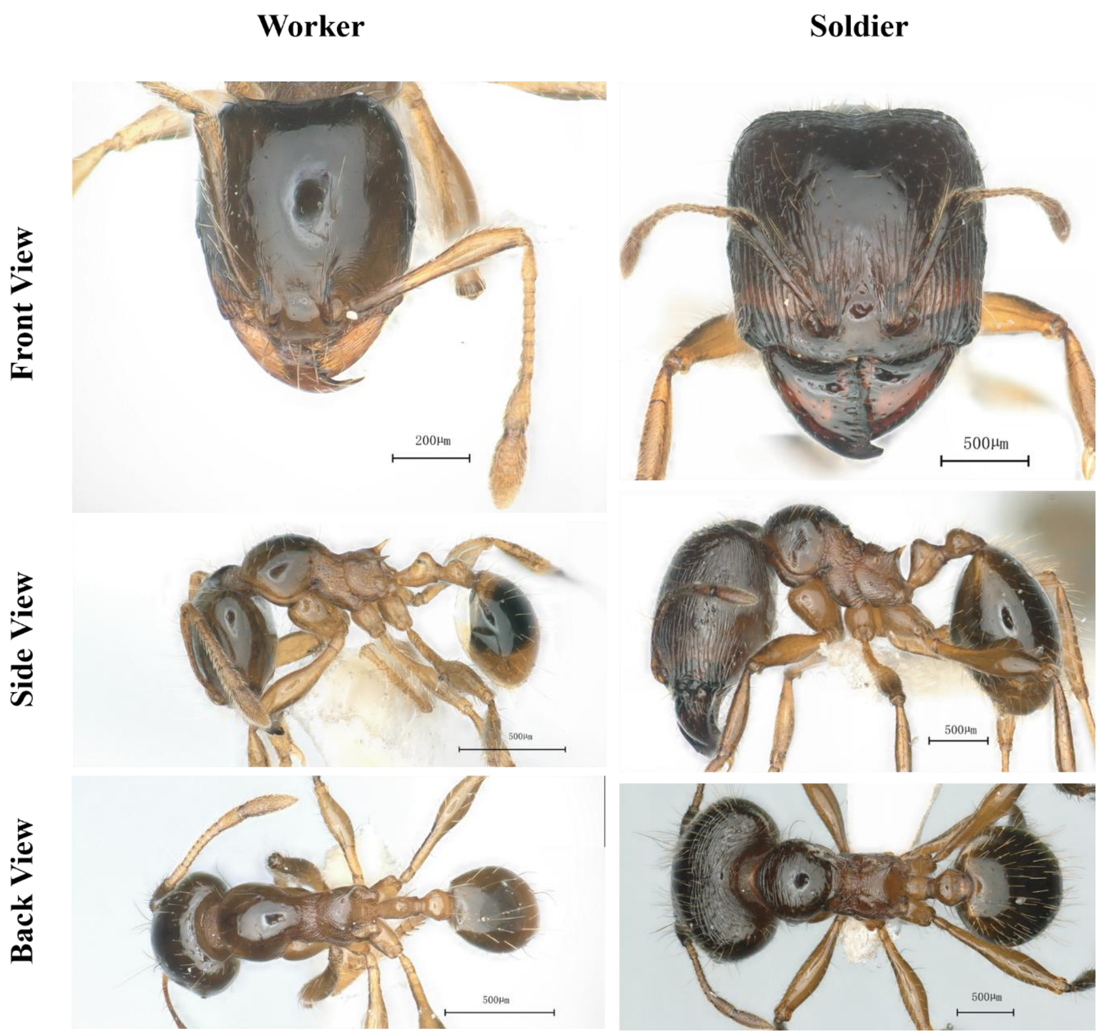

Figure S7. Front view, side view, and vertical view of *Carebara affinis* (Jerdon) (Formicidae: Myrmicinae).

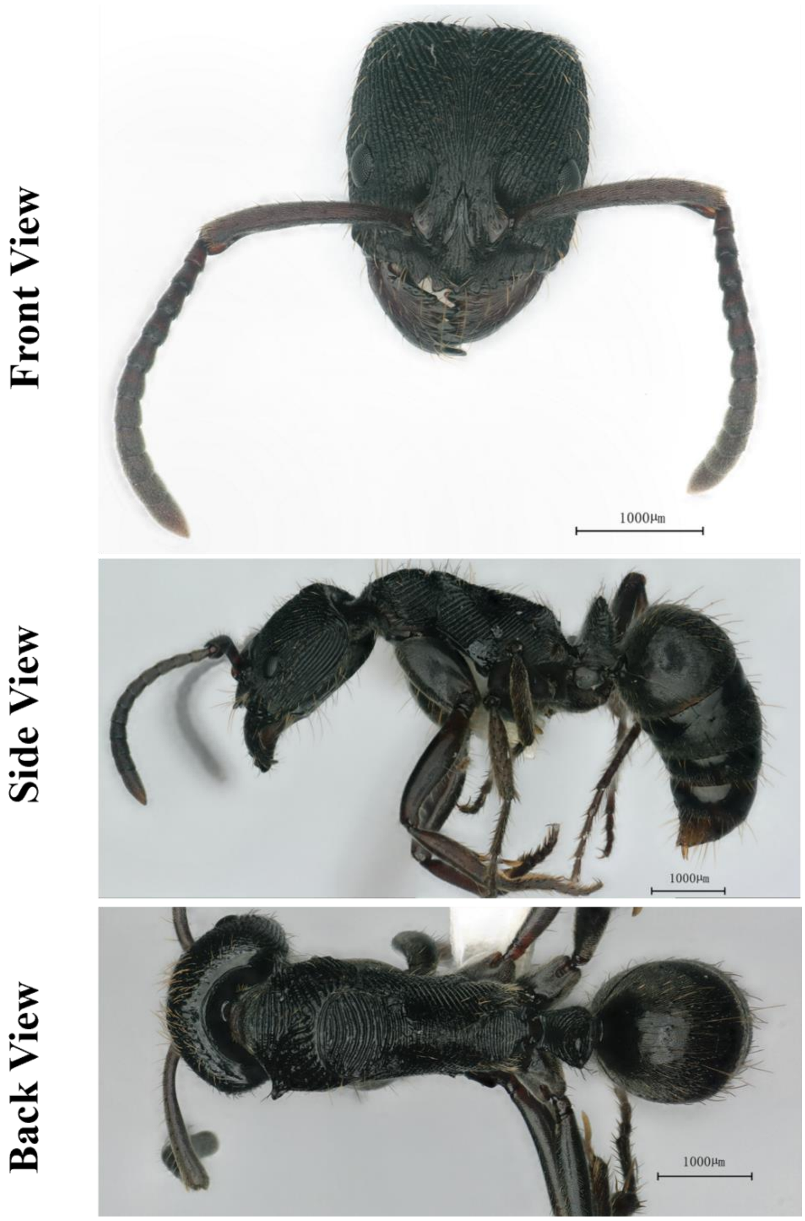

Figure S8. Front view, side view, and vertical view of *Odontoponera transversa* (Smith) (Formicidae: Ponerinae).

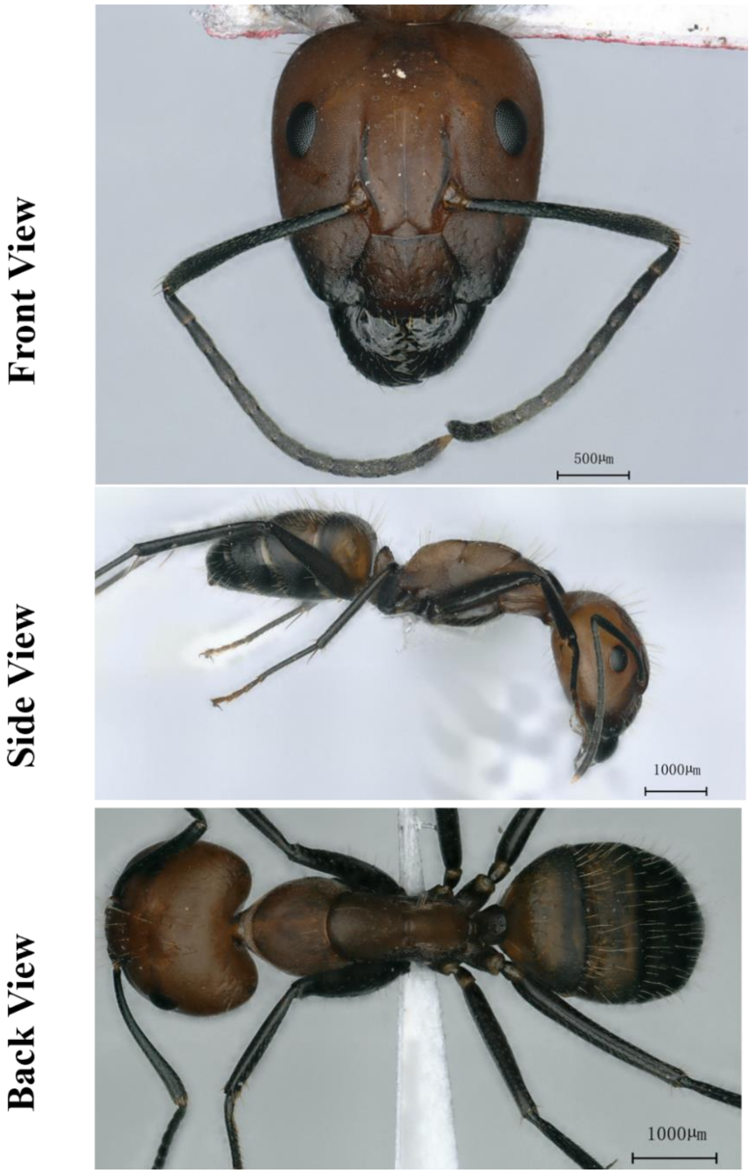

Figure S9. Front view, side view, and vertical view of *Camponotus nicobarensis* Mayr (Formicidae: Formicinae).

Key of ant species that paved adhesive tapes in this study

1 Petiole only (waist consisting of one node) or not obvious …………………………………… 2
- Postpetiole exist (waist consisting of two nodes) ……………………………………………… 4

2 (1). Petiole not obvious, short hairs on thorax, color of antenna is brighter than that of head…………………………………………………………… *Tapinoma melanocephalum*
- Petiole obvious, erect and long hairs on thorax, color of antenna is the same or darker than that of head……………………………………………………………………………… 3

3 (2). Head, thorax, and abdomen were black; obvious striate sculpture on head and thorax……………………………………………………… *Odontoponera transversa*
- Head, thorax, and part of the abdomen were dark orange; smooth body surface……………………………………………………… *Camponotus nicobarensis*

4 (1). 3-segmented apical club……………………………………………… *Pheidole*
- Last two antennal segments enlarged………………………………………………… 5

5 (4). Thorax with a pair of spines ……………………………………… *Carebara affinis*
- Thorax without spine……………………………………………… *Solenopsis invicta*

Figure S10. A key of tape-paving ant species (*Solenopsis invicta*, *Tapinoma melanocephalum*, *Pheidole spp.*, *Carebara affinis*, *Camponotus nicobarensis*, and *Odontoponera transversa*) in this study.
